# Supplementary material for: Computational analysis of 4-1BB-induced NFκB signaling suggests improvements to CAR cell design
Source: Cell Commun Signal. 2022 Aug 26;20:129. doi: 10.1186/s12964-022-00937-w (PMC9413922; doi:10.1186/s12964-022-00937-w)
Supplement: Supplementary file 3 — Additional file 2: Supplementary figures. [file 12964_2022_937_MOESM3_ESM.pdf]

SUPPLEMENTAL FIGURES

Computational analysis of 4-1BB-induced NFκB signaling suggests improvements to CAR cell design

Vardges Tserunyan[1] and Stacey D. Finley[1,2,3]*

[1]Department of Quantitative and Computational Biology, University of Southern California, Los Angeles, CA
[2]Department of Biomedical Engineering, University of Southern California, Los Angeles, CA
[3]Mork Family Department of Chemical Engineering and Materials Science, University of Southern California, Los Angeles, CA

*Corresponding author

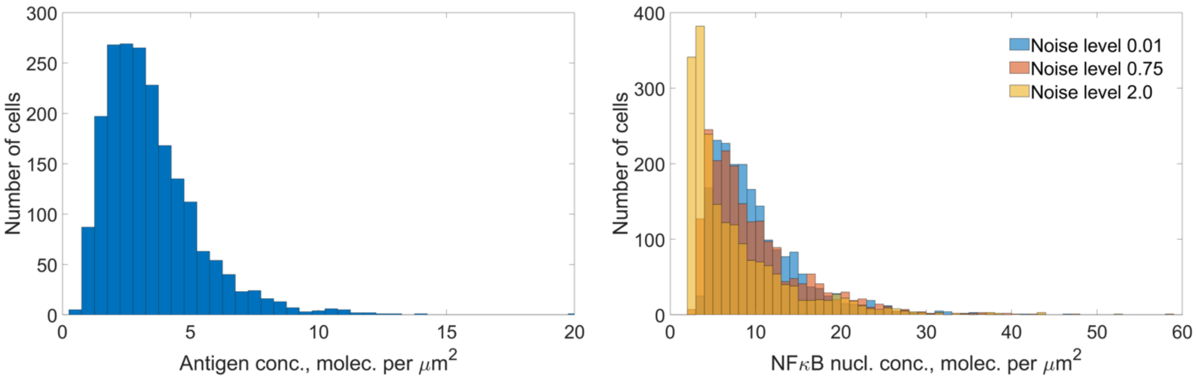

Figure S1: Distribution of antigen concentrations and peak NFκB nuclear concentrations. (A) Histogram of the sampled antigen concentration used throughout Monte Carlo simulations; (B) Histogram of peak NFκB nuclear concentrations observed in response to antigen stimulations from (A) at different intrinsic noise levels.

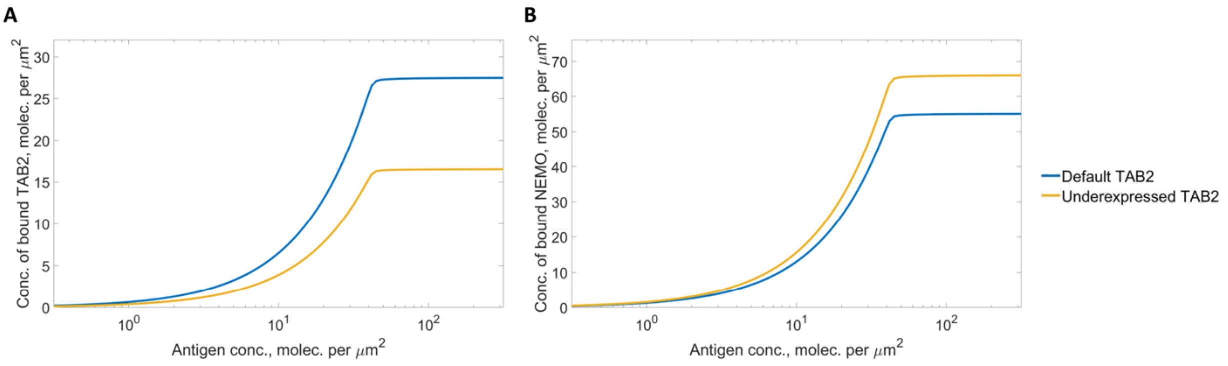

Figure S2: Dose response curves of K-63 Ubiquitin-bound TAB2 and NEMO with default initial concentration or 50% underexpression of TAB2. (A) Concentration of bound TAB2 as a function of antigen concentration; (B) Concentration of bound NEMO as a function of antigen concentration.

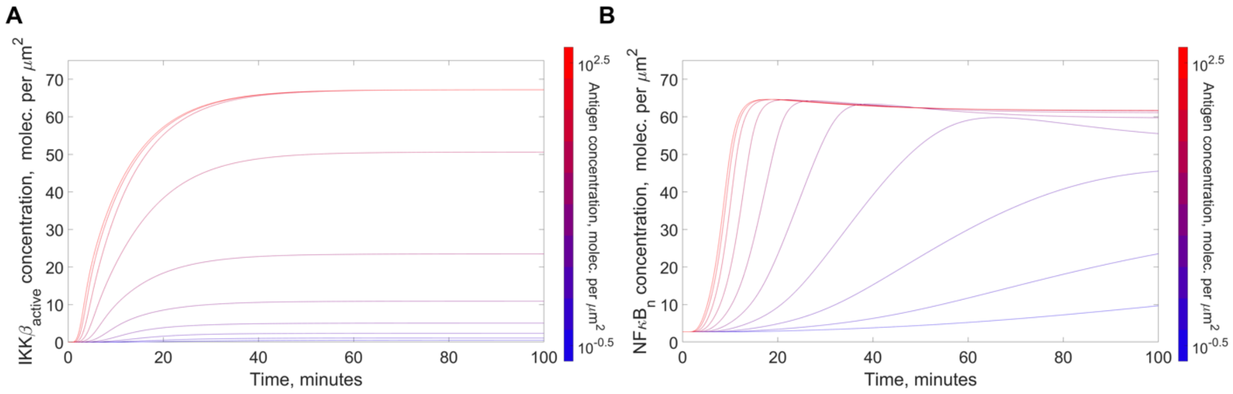

Figure S3: Activation profiles for IKKβ and NFκB in response to antigen binding the CAR with disabled deactivation of IKKβ. The pathway was stimulated with 10 different antigen concentrations *in silico* and activation profiles for IKKβ and NFκB were recorded for each antigen concentration. (A) Concentration of enzymatically active IKKβ; (B) Nuclear concentration of NFκB.

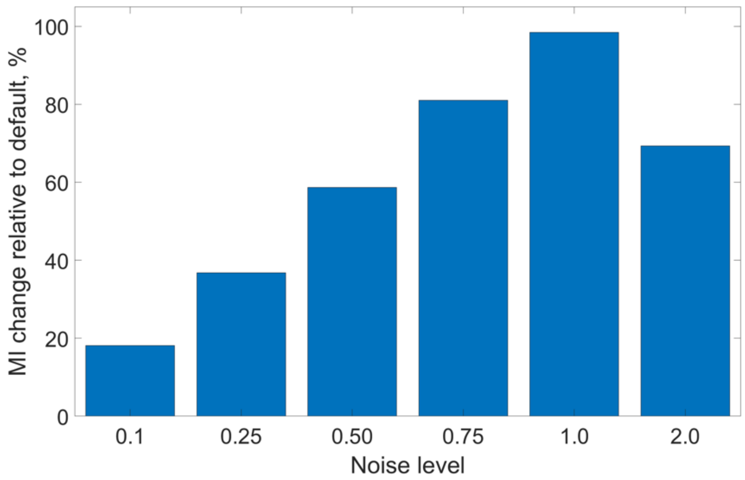

Figure S4: The transduction of information along the canonical NFκB pathway with disabled IKKβ deactivation. Percentages show relative decrease of mutual information at the level of nuclear NFκB concentration compared to the default model at different values of the noise parameter.
